# Grassland Degradation Has Stronger Effects on Soil Fungal Community Than Bacterial Community across the Semi-Arid Region of Northern China

**DOI:** 10.3390/plants11243488

**Published:** 2022-12-13

**Authors:** Congwen Wang, Zhangkai Liu, Wanying Yu, Xuehua Ye, Linna Ma, Renzhong Wang, Zhenying Huang, Guofang Liu

**Affiliations:** 1 State Key Laboratory of Vegetation and Environmental Change, Institute of Botany, Chinese Academy of Sciences, Beijng 100093, China; 2 College of Resources and Environment, University of Chinese Academy of Sciences, Beijng 100049, China; 3 Key Laboratory of Vegetation Ecology of the Ministry of Education, Institute of Grassland Science, Northeast Normal University, Changchun 130024, China

**Keywords:** grassland degradation, soil microbial diversity, microbial community structure, soil properties, semi-arid region

## Abstract

Soil microbes play crucial roles in grassland ecosystem functions, such as soil carbon (C) pool and nutrient cycle. Soil microbes in grasslands are susceptible to the degradation mediated by climate change and anthropogenic disturbance. However, research on how the degradation influences the diversity and community structure of different soil microbial taxa is relatively scarce. We conducted a large-scale field survey to describe the effects of four degradation levels (PD: potential degradation, LD: light degradation, MD: moderate degradation, and SD: severe degradation) on soil bacterial and fungal community in the semi-arid grasslands of northern China. We found that soil moisture, nutrients, and clay content decreased, but soil sand content increased along the increasing degradation gradient. However, the degradation had no effects on soil pH and the C:N ratio. Grassland degradation had non-significant effect on soil bacterial diversity, but it significantly affected soil bacterial community structure. The degradation decreased soil fungal diversity and had a relatively larger influence on the community structure of soil fungi than that of bacteria. The community composition and structure of soil fungi were mainly affected by soil nutrients and texture, while those of soil bacteria were mainly affected by soil pH. These results indicate that changes in soil properties induced by grassland degradation mainly drive the variation in the soil fungal community and have less effect on the soil bacterial community. This study reveals the sensitivity of soil fungal community to grassland degradation, highlighting the priority of soil fungal community for the management and restoration of degraded grasslands.

## 1. Introduction

Grassland is one of the largest terrestrial biomes in the worldwide, and it provides various ecosystem functions and services, such as carbon sequestration, food provision, and livestock support, for supporting human well-being [1]. However, most grasslands have been facing degradation to different degrees [2]. The degradation is a process of retrogressive succession of grasslands, which is mainly manifested by the changes in the plant community structure, biodiversity loss, deterioration of soil habitat, and the decline in plant production [3,4,5]. This harms the health of grassland ecosystems, decreases ecosystem function and stability, and affects human economic development and social stability. 

Climate change and anthropogenic disturbance have been considered as the underlying environmental drivers affecting the structure and function of grassland ecosystems, resulting in grassland degradation [6]. Global warming increases water evapotranspiration of soil surface, aggravates atmospheric drought, and reduces the water input of grassland ecosystems. The extreme drought caused by the change in precipitation pattern also accelerates the course of grassland degradation [2]. In addition, the consequence of overgrazing is the decrease of vegetation coverage and the increase of bare soil area due to excessive foraging and trampling of livestock [7,8]. In the process of grassland degradation, soil physicochemical properties may be altered significantly [9,10]. Some studies found that, with the increase in the gradation degree, soil moisture and nutrients gradually decreased, and soil texture changed significantly, resulting in changes in the composition and structure of plant communities [5,11,12,13]. These changes in soil properties and the composition and structure of aboveground plant communities would have an adverse influence on the soil microbial community, and further impact ecosystem functions and services.

Soil microbes are important drivers of terrestrial ecosystem processes and play important roles in soil formation, biogeochemical cycle and ecosystem stability [14,15,16]. Some studies have shown the responses of soil microbial diversity and community structure to grassland degradation [3,5,17,18,19]. It can affect soil microbial community via multiple pathways, such as plant community and soil attributes [20]. The changes in plant biomass, diversity, and community structure induced by the degradation had strong influences on the soil microbial community [3,17,21]. Decreased plant biomass induced by grassland degradation reduces the quantity of litter and root excretion input to soils; this affects the substrate availability of soil microbes [22,23,24]. The decrease in plant diversity mediated by the degradation would reduce soil microbial diversity by reducing substrate diversity of saprophytic microbes and host diversity of symbiosis and pathogenic microbes [15]. 

Compared to the biotic influences of the aboveground plant community, the various soil physicochemical indices also regulate the composition and structure of soil bacterial and fungal communities. Soil bacterial diversity was mainly regulated by soil pH [25,26], and soil fungal diversity was mainly controlled by soil nutrients and texture [27,28]. Grassland degradation reduced soil nutrients and altered soil texture, decreasing soil fungal diversity [29]. A study from the alpine steppe of the Qinghai-Tibetan Plateau has shown that the degradation significantly changed the soil bacterial community structure, but it did not affect soil bacterial diversity [30]. In the desert steppe, the degradation drastically shifted soil bacterial community structure due to the changes in soil attributes, such as soil moisture, pH, and sand content [11]. Zhao et al. [29] reported that grassland degradation changed the soil bacterial and fungal community structure, and soil bacterial diversity showed unimodal pattern with the increase of degradation degree, while soil fungal diversity significantly decreased. Therefore, the changes in soil attributes induced by grassland degradation have inconsistent effects on soil bacterial and fungal communities [13,17,31]. Uncovering the effects and mechanisms of grassland degradation on soil microbial diversity and community structure will provide an insightful understanding on the conservation and ecological restoration of grassland ecosystems.

Semi-arid grassland in northern China is an important green ecological barrier and is the basis of husbandry development. Grassland in the semi-arid region is very fragile and sensitive to climate change and anthropogenic disturbance [32,33]. In recent years, mean annual temperature in this region gradually increased, precipitation patterns have changed, and the events of extreme precipitation and drought become more frequent [34]. In addition, due to the low carrying capacity of husbandry of natural grassland in the semi-arid region of northern China, there is a serious shortage of forage in winter, and overgrazing has a significant adverse influence on the fragile ecological environment of the region [35,36]. Therefore, under the joint influence of climate change and anthropogenic disturbance, ecological environment of semi-arid grassland has been deteriorating, which seriously affects the ecosystem security of this region and restricts the sustainable development of husbandry. However, studies on soil microbial community composition and structure during grassland degradation mainly focus on a specific soil microbial taxon, i.e., bacteria or fungi, and they are relatively scarce on soil bacterial and fungal community at large spatial scales. In order to uncover the influences of grassland degradation on soil properties and the relative contribution of the degradation on soil bacterial and fungal community, we test the following hypotheses: (1) grassland degradation would have adverse influences on soil physical and chemical attributes; (2) grassland degradation would have a stronger effect on soil fungi than on soil bacteria. In this study, we investigated the soil bacterial and fungal community of 54 plant communities with different degradation levels at 18 locations across the semi-arid grasslands of northern China and described how grassland degradation affected soil properties and the diversity and community structure of soil bacterial and fungal community.

## 2. Results

### 2.1. Effects of Grassland Degradation on Plant and Soil Properties

**Figure 1:**
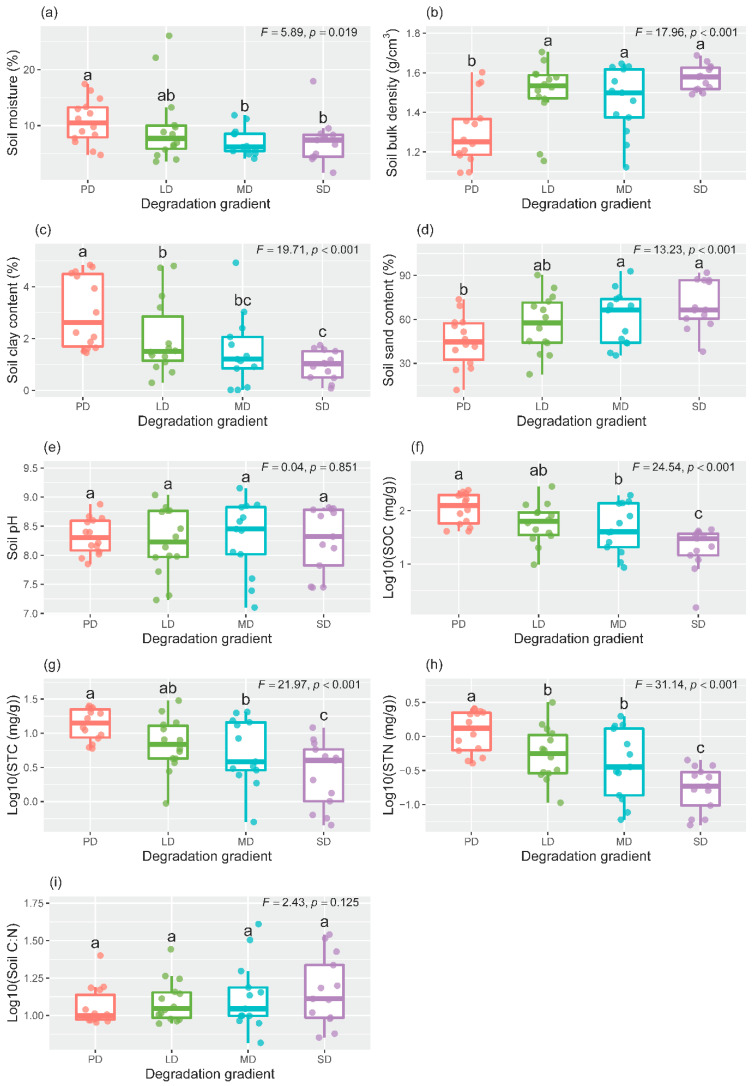
The effects of grassland degradation on soil moisture (**a**), bulk density (**b**), clay content (**c**), sand content (**d**), pH (**e**), soil organic carbon (SOC; (**f**)), soil total carbon (STC; (**g**)), soil total nitrogen (STN; (**h)**), and soil C:N (**i**). Different lowercase letters represent significant differences among the degradation levels at *p* < 0.05. PD, potential degradation; LD, light degradation; MD, moderate degradation; SD, severe degradation.

Along the degradation gradient, vegetation coverage and plant species richness significantly decreased (Figure S1). For soil physical properties, soil moisture and soil clay content significantly decreased along the degradation gradient (Figure 1a,c), while soil sand content and soil bulk density significantly increased (Figure 1b,d). For soil chemical properties, grassland degradation decreased soil organic carbon (SOC), soil total carbon (STC), and soil total nitrogen (STN), however, the degradation had non-significant effects on the soil C:N ratio and pH (Figure 1e,i).

### 2.2. Effects of Grassland Degradation on Diversity and Community Structure of Soil Bacteria and Fungi

Across all the sites, the dominant phyla of soil bacteria were Proteobacteria, Actinobacteria, Acidobacteria, Chloroflexi, Gemmatimonadetes, and Bacteroidetes, and their relative abundances were greater than 90% (Table S1). The dominant phyla of soil fungi were Ascomycota, Basidiomycota, Mortierellomycota, Glomeromycota, and Chytridiomycota, and their relative abundances were greater than 85% (Table S2).

**Figure 2:**
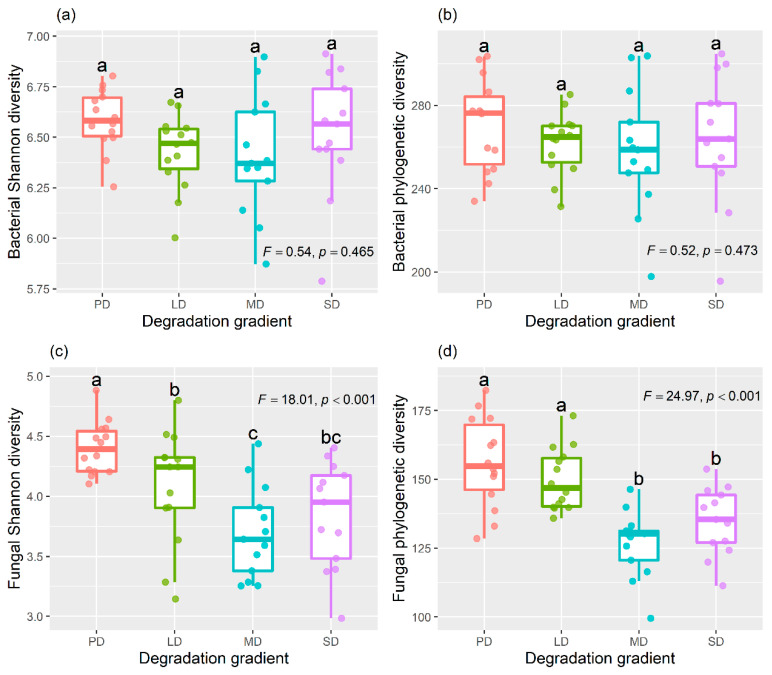
The effects of grassland degradation on the soil bacterial Shannon diversity (**a**) and phylogenetic diversity (**b**), and soil fungal Shannon diversity (**c**) and phylogenetic diversity (**d**). Different lowercase letters represent significant differences among the degradation levels at *p* < 0.05. PD, potential degradation; LD, light degradation; MD, moderate degradation; SD, severe degradation.

One-way analysis of variance (ANOVA) and least significant difference (LSD) multiple comparison showed that soil bacterial Shannon diversity and phylogenetic diversity did not significantly differ among the four degradation levels (Figure 2a,b). However, soil fungal Shannon diversity significantly decreased along the degradation gradient, and soil fungal phylogenetic diversity was significantly lower in MD and SD than in PD and LD (Figure 2c,d).

**Figure 3:**
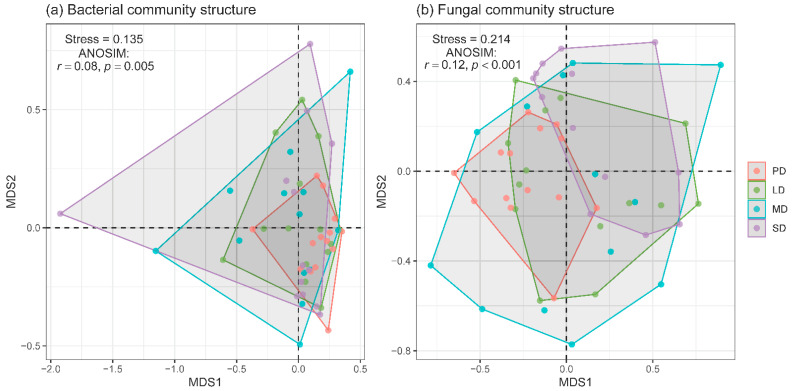
Non-metric multidimensional scaling (NMDS) ordination plots derived from the Bray-Curtis distance matrix of soil bacterial (**a**) and fungal (**b**) communities. PD, potential degradation; LD, light degradation; MD, moderate degradation; SD, severe degradation.

The non-metric multidimensional scaling (NMDS) and analysis of similarities (ANOSIM) showed that there were significant differences in the community structure of both soil bacteria and fungi among the four degradation levels (Figure 3). The community structures of soil bacteria and fungi were changed in MD and SD compared to those in PD; the difference in community structure between LD and SD was significant for soil fungi but not for soil bacteria (Table 1). In addition, grassland degradation had stronger effects on the community structure of soil fungi than that of soil bacteria (Figure 3; Table 1).

### 2.3. Relationships between Plant or Soil Properties and Soil Microibal Community

**Figure 4:**
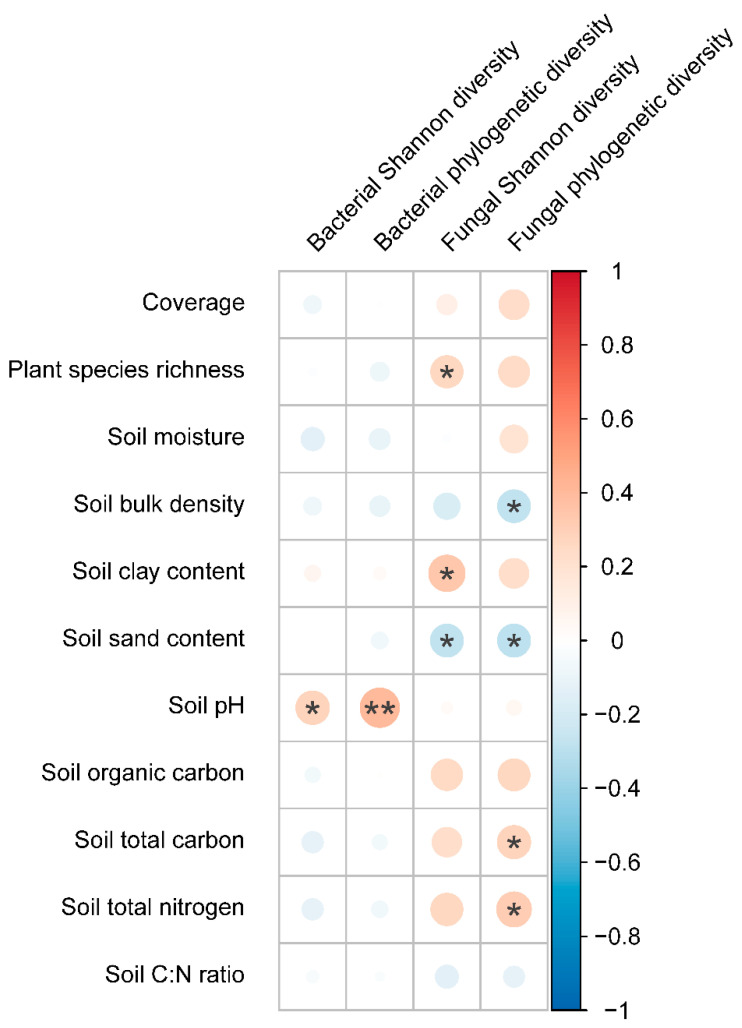
Correlation plot showing the relationships between soil bacterial and fungal diversity (Shannon diversity and phylogenetic diversity) and plant and soil attributes. The circle size denotes the correlation coefficients. Red and blue circles denote positive and negative correlations, respectively. *, *p* < 0.05, **, *p* < 0.01.

The Pearson correlation analysis showed that soil bacterial Shannon diversity and phylogenetic diversity were significantly and positively correlated with soil pH, and had non-significant relationships with vegetation coverage, plant species richness, and other soil attributes (Figure 4). Soil fungal Shannon diversity had significant and positive relationships with plant species richness and soil clay content and had significant and negative relationship with soil sand content. Additionally, soil fungal phylogenetic diversity was negatively related to soil sand content and soil bulk density, and positively related to STC and STN (Figure 4).

**Figure 5:**
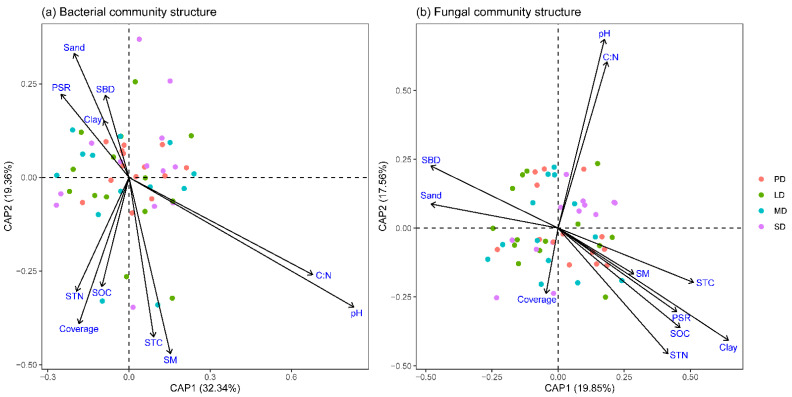
Constrained analysis of proximities (CAP) of soil bacterial (**a**) and fungal (**b**) community structure. PD, potential degradation; LD, light degradation; MD, moderate degradation; SD, severe degradation; SM, soil moisture; SOC, soil organic carbon; STC, soil total carbon; STN, soil total nitrogen; SBD, soil bulk density; Clay, soil clay content; Sand, soil sand content; C:N, soil C:N ratio; PSR, plant species richness.

The constrained analysis of proximities (CAP) showed that the first and second axes explained 32.34% and 19.36% of the total variance in soil bacterial community structure, respectively, in which soil pH, C:N ratio, and soil moisture were the dominant factors (Figure 5a). The CAP showed that the first and second axes explained 19.85% and 17.56% of the total variance in the soil fungal community structure, respectively, in which soil sand content, soil bulk density, soil clay content, STC, SOC, STN, and plant species richness were the dominant influential factors (Figure 5b).

## 3. Discussion

Grassland degradation is a common ecological problem in grassland ecosystems worldwide. In the present study, we described the effects of grassland degradation on soil properties and soil bacterial and fungal communities in the semi-arid grasslands of northern China and revealed the drivers of the diversity and community structure of soil bacteria and fungi. 

### 3.1. Grassland Degradation Affects Soil Attributes

We found that the degradation significantly altered soil physicochemical properties in the semi-arid grasslands of northern China. With the aggravation of degradation, soil moisture decreased continuously (Figure 1a), which is consistent with other studies from grassland ecosystems [5,37]. In the alpine steppe of the Tibetan Plateau, grassland degradation significantly decreased soil moisture, from 10% of natural grasslands to 2.6% of degraded grasslands with the extremely severe degradation [5]. Grassland degradation caused the decrease of vegetation coverage and the increase of bare land area, which resulted in the increase in soil water evaporation and the reduction in soil water-retention capacity [7]. In addition, grassland degradation led to the decrease in soil clay content, the increase in soil sand content and bulk density (Figure 1c,d). Wind erosion is a crucial driver in the degradation process of semi-arid grasslands; it has a mechanical sorting process for the particle size on the soil surface [38]. In the semi-arid region of northern China, strong winds often occur in winter and early spring [39]. The low vegetation coverage in degraded grasslands aggravated the effect of wind erosion on the top soil. Lighter clay and silt particles in the soil surface were transported by the wind to other places, resulting in the coarsening of the soil texture. The increase in coarse particles was not conducive to maintaining soil moisture, and this is also one of the reasons that grassland degradation reduced soil moisture. We acknowledged that soil moisture data from one sampling time could not accurately reflect soil water condition for a long-term period. Intensive soil water monitoring across multiple geographical sites should be encouraged in future to improve the accuracy of assessing the potential effects of vegetation structure on soil hydraulic processes. 

Grassland degradation may affect soil chemistry via direct or indirect pathways. However, we found that grassland degradation had no effect on soil pH (Figure 1e). Similarly, grassland degradation did not change soil pH in the alpine grassland of the Qilian Mountain China [40]. However, Fan et al. [11] found that degradation significantly increased the soil pH of the desert steppe. Therefore, the impact of grassland degradation on soil pH may vary across different grassland types. Furthermore, with the increase in grassland degradation, SOC, STC and STN showed decreasing trends, while the soil C:N ratio did not change (Figure 1f,i). Other studies have reported similar results [11,41]. The previous study also showed that grassland degradation can decrease soil nutrients by changing soil texture [42]. In the process of degradation, the decrease of vegetation coverage will reduce the protection for soil nutrients, and the decrease of grassland productivity will also reduce the input of soil nutrients from plant litters or root exudates. In addition, the reduction of soil clay content and resulting coarsening of soil particles mediated by grassland degradation will lead to the decrease of soil nutrients [5]. The reason may be that most of the soil organic matter is combined with fine soil particles, such as soil clay. Overall, these results support our first hypothesis that grassland degradation would have adverse influences on soil physical and chemical attributes. Therefore, long-term monitoring of the changes in sensitive soil physicochemical properties in response to the degradation gradient, such as soil clay content or bulk density, and soil C and N contents, aids in uncovering the risk of grassland degradation in the early stage of degradation.

### 3.2. Effects of Grassland Degradation on Soil Bacterial Community

As one of the important components of terrestrial ecosystems, soil microbes can control multiple ecological processes, such as carbon cycle and nutrient turnover, and are important drivers of material circulation and energy flow [14,43,44]. Our study found that grassland degradation had a non-significant effect on Shannon diversity and the phylogenetic diversity of soil bacteria (Figure 2a,b). However, a study from the alpine steppe of Tibetan Plateau found that grassland degradation increased soil bacterial diversity because grassland degradation increased the soil C:N ratio, which was positively correlated with soil bacterial diversity [5]. In this study, we found that soil bacterial diversity was less affected by the soil C:N ratio. In addition, our results showed that soil pH had strong effects on soil bacterial Shannon diversity and phylogenetic diversity (Figure 4), however grassland degradation had no effect on soil pH (Figure 1e), which may be one of the reasons that grassland degradation did not affect soil bacterial diversity. 

While grassland degradation had non-significant effect on soil bacterial diversity, the degradation significantly changed the soil bacterial community structure, particularly in MD and SD (Figure 3a, Table 1). The finding was consistent with the study from a desert steppe [11], which suggested that the soil bacterial community structure diffed among various degradation levels, and the main driver was soil pH. Our study found that the main factors affecting the soil bacterial community structure in degraded grassland were soil pH, C:N ratio, and soil moisture (Figure 5a). While grassland degradation had non-significant effects on soil pH and the C:N ratio, soil moisture decreased significantly along the degradation gradient, which was one of the reasons that grassland degradation changed the soil bacterial community structure. In general, grassland degradation had little effects on the composition and structure of soil bacterial communities in the semi-arid grasslands of northern China.

### 3.3. Effects of Grassland Degradation on Soil Fungal Community

Fungi, as one of the important taxa of soil microbes, can form a symbiosis with plant roots and improves the nutrient utilization efficiency of plants [45]. Soil fungi participates in the decomposition of plant litter and residues and is one of the indispensable drivers of carbon and nutrient cycling in soils [46]. In this study, we found that both the Shannon diversity and phylogenetic diversity of soil fungi decreased with the grassland degradation (Figure 2c,d). Phylogenetic diversity is a biodiversity measure based on evolutionary relationships between species and is a proxy for evolutionary potential [47]. The decrease of phylogenetic diversity induced by grassland degradation would reduce the specialized fungal taxa and fungal evolutionary potential in soils. In addition, we found that soil fungal diversity was mainly affected by plant species richness, soil nutrients and texture (Figure 4). The diversity of litter quality and root exudations mediated by plant diversity can provide more belowground resource niches for soil fungi, implying that higher plant diversity contributes to increasing soil fungal diversity [22]. Thus, the decrease of plant diversity induced by grassland degradation deceased soil fungal diversity. Furthermore, microbial metabolism was limited by nutrients in arid and semi-arid regions [48]. The previous study also found that soil fungal diversity had significant and positive relationship with soil nutrients [49]. For the reasons above, the decrease of soil nutrients mediated by grassland degradation significantly reduced soil fungal diversity. Meanwhile, the change in soil texture induced by grassland degradation had a significant effect on soil fungal diversity [28]. The soil texture indirectly affected soil fungal diversity by regulating soil nutrients, e.g., the decrease of soil clay content and the increase of soil sand content associated with poor soil quality or fertility. 

We also found that grassland degradation had a significant effect on the soil fungal community structure (Figure 3b, Table 1). A study from the meadow steppe also showed a similar finding [17]. In addition, grassland degradation had a larger impact on the community structure of soil fungi than that of soil bacteria (Figure 3; Table 1). The dominant drivers of the soil fungal community structure were soil nutrients, texture, and plant diversity (Figure 5b), which were changed by grassland degradation (Figure 2 and Figure S1). Additionally, 70–90% of terrestrial plants are infected by mycorrhizal fungi [50], and the infected mycorrhizas can improve the utilization efficiency of organic matter and nutrient elements for plants [45]. Based on the strong interaction mechanism between plant and soil fungi, we found that plant species richness had more influence on the fungal community structure than that of the bacterial community structure.

Overall, grassland degradation had stronger effects on the composition and structure of soil fungal community than those of the soil bacterial community, emphasizing the vulnerability and sensitivity of soil fungi to grassland degradation, and supporting our second hypothesis that grassland degradation would have a stronger effect on soil fungi than on soil bacteria. Therefore, soil fungi should be considered in ecological restoration practices of degraded grasslands. Covering or incorporating litter substrate, or planting herbaceous plants with relatively abundant endophytic fungi and arbuscular mycorrhiza fungi can ameliorate soil fungal abundance and composition in degraded grasslands. Since there was strong coordination between soil microbial diversity and plant diversity [22,51], altering soil microbes, especially soil fungi in ecological restoration, aids to improving ecosystem functions and services of degraded grassland via the positive feedback between above and belowground biodiversity. Additionally, integrating plant and soil microbial community attributes in grassland restoration assessment is conductive to comprehensively revealing the restoration status of grassland from above and below-ground ecological processes.

## 4. Materials and Methods 

### 4.1. Study Region and Sample Collection

This study was conducted in the semi-arid grasslands of northern China, spanning Jilin Province, Inner Mongolia Autonomous Region, and Ningxia Hui Autonomous Region (Figure S2). This region has a temperate continental climate and the mean annual temperature ranges from 0.8 to 9.0 °C; the mean annual precipitation ranges from 245 to 494 mm (Worldclim) [52]. Along the large-scale transect from northeast to northwest, we selected 18 locations for the field investigation and sampling, and selected three plant communities with different dominant plant species presenting degradation to some extent in each location during the peak growing season in July and August 2018. According to bare land area and vegetation coverage compared to natural vegetation in each location [5,53], 54 plant communities were divided into four degradation levels, including potential degradation (PD, 10% less than natural vegetation coverage; 14 communities), light degradation (LD, 10–20% less than natural vegetation coverage; 14 communities), moderate degradation (MD, 20–50% less than natural vegetation coverage; 13 communities), and severe degradation (SD, 50% less than natural vegetation coverage; 13 communities). For plant community with only herb layer (33 sites), we set three 1 × 1 m^2^ quadrats to conduct plant community survey (vegetation coverage, plant species composition and abundance) within an area of 1000 m^2^. For plant community with shrub and herb layers, three 5 × 5 m^2^ and three 1 × 1 m^2^ quadrats were chosen to conduct a plant community survey of shrub and herb layers, respectively, within an area of 1000 m^2^. Soil samples were collected from three corresponding plant quadrats within each plant community. Soil cores were collected in a depth of 10 cm with a cylindrical soil sampler (5 cm diameter) and immediately processed using a 2 mm sieve to remove roots, debris and gravel, then soil samples were placed into sterile plastic bags. Samples for soil microbial community analysis were stored in a refrigerator at −20 °C, and the other for measuring soil properties were air-dried and stored.

### 4.2. Soil Properties Measurement

Soil moisture (SM, %) was determined using the drying method, and each soil sample was measured after drying at 105 °C for 48 h. Soil pH was measured by the potentiometry, using a pH meter (PB-10, Sartorius, Germany) in a 1:2.5 (soil:water) suspension. Soil bulk density (SBD, g/cm^3^) was measured by the ring knife method, and soil samples were collected using a 100 cm^3^ ring knife and measured after drying at 105 °C for 48 h. Soil clay, silt, and sand content (%) was measured using a laser particle analyzer (Mastersizer 2000, Malvern, England). Soil organic carbon (SOC, mg/g) was determined by the potassium dichromate oxidation heating method. Elemental analyzer (Vario EL Cube CHNOS Elemental Analyzer, Elementar Analysensysteme GmbH, Germany) was used to measure soil total carbon (STC, mg/g) and total nitrogen (STN, mg/g). The soil C:N ratio was calculated as STC divided by STN.

### 4.3. DNA Extraction and Pyrosequencing

Deoxyribonucleic acid (DNA) was extracted from 0.5 g of each soil sample using a Power Soil DNA Isolation Kit (MoBio Laboratories, Carlsbad, CA, USA) following the manufacturer’s instructions. Bacterial 16S ribosomal RNA (16S) genes and fungal internal transcribed spacer (ITS) regions were amplified via polymerase chain reaction (PCR) using the primer pairs 338F/806R [54] and ITS1F/ITS2 [55] combined with adapter sequences and barcode sequences, respectively. The PCR mixture (25 µL) containing 12.5 µL 2× Taq PCR MasterMix, 3 µL bovine serum albumin (2 ng/µL), 2 µL primers (5 µM), 2 µL template DNA, and 5.5 µL double-distilled H_2_O (ddH_2_O). The PCR amplification program included initial denaturation at 94 °C for 5 min, followed by 28 and 32 cycles for 16S and ITS, respectively, of 94 °C for 30 s, 55 °C for 30 s and 72 °C for 60 s with a final extension at 72 °C for 7 min. The PCR products were purified using a QIAquick gel extraction kit (QIAGEN, Germany), and sequenced on an Illumina MiSeq 300 PE platform (Illumina, San Diego, CA, USA) according to the protocol.

### 4.4. Bioinformatics Analysis 

Single reads were assigned to samples based on their unique barcode and truncated by cutting off the barcode and primer sequence. Quality filtering of raw reads was performed under specific filtering conditions to obtain high-quality clean reads using QIIME [56]. The UCHIME algorithm [57] was used to remove chimeric sequences based on the SILVA [58] and UNITE [59] databases for bacteria and fungi, respectively. The sequences were clustered into operational taxonomic units (OTU) at a similarity level of 97% using UPARSE [60]. Taxonomy was identified for each OTU using the RDP Classifier algorithm [61] trained on the SILVIA [58] and UNITE [59] databases for bacterial and fungal sequences. In order to assess bacterial and fungal community at the same sequencing depth, the datasets of OTU were subsampled to 13,182 sequences for bacteria and 17,642 sequences for fungi (Figure S3). Soil bacterial and fungal Shannon diversity were calculated using the *vegan* package [62] in R software (v3.5.3) [63] based on OTU tables of bacteria and fungi. We also calculated Faith’s phylogenetic diversity of bacteria and fungi using the *picante* package [64]. 

### 4.5. Statistical Analysis

SOC, STC, STN, and the soil C:N ratio were log10-transformed to satisfy the assumption of the normal distribution. As there was an extremely significant negative relationship between soil silt content and soil sand content (*r* = 0.998, *p* < 0.001; Figure S4), soil silt content was excluded in all data analyses. One-way analysis of variance (ANOVA) followed by least significant difference (LSD) multiple comparisons was used to detect the differences in vegetation coverage, plant species richness, soil properties, soil bacterial and fungal diversity (Shannon diversity and phylogenetic diversity) among different degradation levels. We used Pearson correlation analysis to determine the relationships between soil bacterial and fungal diversity and plant and soil attributes. Soil bacterial and fungal community structure among different degradation levels were characterized by non-metric multidimensional scale (NMDS) analysis based on Bray-Curtis distance, and the differences in soil microbial community structure among different degradation levels were tested by analysis of similarities (ANOSIM). Constrained analysis of proximities (CAP) was used to examine the main factors driving soil bacterial and fungal community structure, in which degradation gradient was taken as a constrained factor. The above statistical analyses and graphics were done using the *stats*, *corrplot*, *vegan*, and *ggplot2* packages of R software (v3.5.3) [64], and the significance level of all statistical tests was set at *p* < 0.05.

## 5. Conclusions

Grassland degradation has adverse influences on soil environments, such as decreasing soil moisture, nutrients and increasing soil sand content and soil bulk density. Furthermore, grassland degradation had no effect on soil bacterial Shannon and phylogenetic diversity, while it reduced soil fungal Shannon and phylogenetic diversity. Additionally, the degradation had relatively stronger influences on the soil fungal community structure than that of the soil bacterial community structure. The changes in the diversity and community structure of soil fungi in response to grassland degradation owe to soil nutrients and texture. These findings suggest that grassland degradation had stronger effect on soil fungal community than soil bacterial community. These emphasize the vulnerability and sensitivity of the soil fungal community to grassland degradation, highlighting the priority of the soil fungal community for the management and restoration of degraded grasslands.

## Figures and Tables

**Table 1:** Analysis of similarity (ANOSIM) of soil bacterial and fungal community structure between different degradation levels. The bold values represent the significant difference between different degradation levels at *p* < 0.05.

	Soil Bacterial Community Structure				Soil Fungal Community Structure			
	PD	LD	MD	SD	PD	LD	MD	SD
PD		0.312	**0.002**	**<0.001**		0.067	**0.001**	**<0.001**
LD	0.011		0.391	0.075	0.076		0.182	**0.043**
MD	**0.172**	0.004		0.571	**0.200**	0.038		0.371
SD	**0.234**	0.062	0.013		**0.340**	**0.086**	0.009	

Note: the values in the lower triangle represent the magnitude of difference between degradation levels, and the values in the upper triangle represent *p* values. PD, potential degradation; LD, light degradation; MD, moderate degradation; SD, severe degradation.

## Data Availability

The raw data sequences of soil microbes in this study have been submitted in SRA of NCBI database and the BioProject accession numbers are PRJNA724862 and PRJNA724863 for bacteria and fungi, respectively.

## Supplementary Materials

The following supporting information can be downloaded at https://www.mdpi.com/article/10.3390/plants11243488/s1, **Table S1**. Relative abundance of soil bacteria at phylum level in all soil bacterial communities and differences among grassland degradation levels. The phylum with relative abundance of less than 1% were categorized to “Others”; **Table S2.** Relative abundance of soil fungi at phylum level in all soil bacterial communities and differences among grassland degradation levels. The phylum with relative abundance of less than 1% were categorized to “Others”; **Figure S1.** The effects of grassland degradation on community coverage (a) and plant species diversity (b). Different lowercase letters represent significant differences among the degradation gradients at *p* < 0.05. PD, potential degradation; LD, light degradation; MD, moderate degradation; SD, severe degradation; **Figure S2.** Map of study region and sampling locations across the semi-arid grasslands of northern China; **Figure S3.** The rarefaction curves of soil bacterial (a) and fungal (b) samples across 54 sites in semi-arid grasslands of northern China; **Figure S4.** The relationship between soil silt content and soil sand content. The red line represents significant correlation at *p* < 0.05.
