# Supplementary material for: Grassland Degradation Has Stronger Effects on Soil Fungal Community Than Bacterial Community across the Semi-Arid Region of Northern China
Source: Plants (Basel). 2022 Dec 13;11(24):3488. doi: 10.3390/plants11243488 (PMC9782112; doi:10.3390/plants11243488)
Supplement: Supplementary file 1 [file plants-11-03488-s001.zip › plants-2033480-supplementary.pdf]

Supplementary materials

Table S1 Relative abundance of soil bacteria at phylum level in all soil bacterial communities and differences among grassland degradation levels. The phylum with relative abundance of less than 1% were categorized to “Others”.

Phylum (%)	All	PD	LD	MD	SD
	Mean±SE	Mean±SE	Mean±SE	Mean±SE	Mean±SE
Proteobacteria	26.07±0.74	25.3±1.19	27.35±1.43	25.18±1.81	26.4±1.59
Actinobacteria	24.28±0.82	26.16±1.12	25.42±2.01	22.3±1.56	22.99±1.66
Acidobacteria	18.58±1.45	16.71±1.93	17.06±2.35	23.14±3.87	17.68±3.24
Chloroflexi	8.85±0.25	8.71±0.26	8.26±0.43	8.91±0.72	9.59±0.48
Gemmatimonadetes	6.93±0.51	7.16±0.48	6.71±0.78	6.2±1.34	7.66±1.37
Bacteroidetes	5.51±0.28	5.85±0.56	5.07±0.49	5.11±0.59	6.01±0.62
Firmicutes	1.48±0.15	1.27±0.17	2.13±0.37	0.89±0.12	1.61±0.34
Nitrospirae	1.47±0.09	1.71±0.25	1.48±0.15	1.32±0.14	1.33±0.16
Cyanobacteria	1.3±0.31	1.29±0.38	1.83±1.12	1.01±0.26	1.03±0.24
Verrucomicrobia	1.28±0.1	1.68±0.2	1.1±0.15	1.34±0.27	0.98±0.15
Others	4.25±0.15	4.17±0.18	3.61±0.2	4.59±0.33	4.69±0.37

Note: SE, standard error; PD, potential degradation; LD, light degradation; MD, moderate degradation; SD, severe degradation.

Table S2 Relative abundance of soil fungi at phylum level in all soil bacterial communities and differences among grassland degradation levels. The phylum with relative abundance of less than 1% were categorized to “Others”.

Phylum (%)	All	PD	LD	MD	SD
	Mean±SE	Mean±SE	Mean±SE	Mean±SE	Mean±SE
Ascomycota	60.23±1.54	58.08±2.68	58.93±2.66	64.33±3.24	59.85±3.76
Basidiomycota	15.47±1.08	19.58±1.66	13.58±1.93	14.42±2.23	14.13±2.62
Unidentified	10.76±0.84	8.79±1.26	11.39±1.7	12.6±2.34	10.35±1.27
Mortierellomycota	6.36±0.78	7.55±0.89	8.94±2.49	3.44±0.54	5.2±1.04
Glomeromycota	3.34±0.39	4.14±0.89	2.7±0.58	3.05±0.79	3.45±0.88
Chytridiomycota	2.29±0.41	1.03±0.18	2.21±0.58	1.76±0.51	4.27±1.36
Others	1.59±0.53	0.93±0.18	2.13±1.64	0.56±0.19	2.74±1.29

Note: SE, standard error; PD, potential degradation; LD, light degradation; MD, moderate degradation; SD, severe degradation.

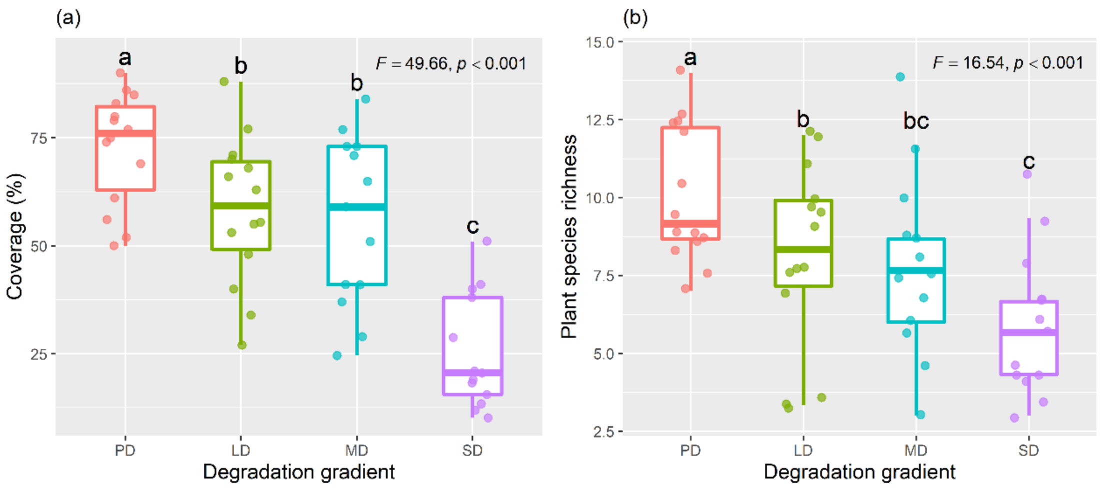

Figure S1 The effects of grassland degradation on community coverage (a) and plant species diversity (b). Different lowercase letters represent significant differences among the degradation gradients at $p < 0.05$. PD, potential degradation; LD, light degradation; MD, moderate degradation; SD, severe degradation.

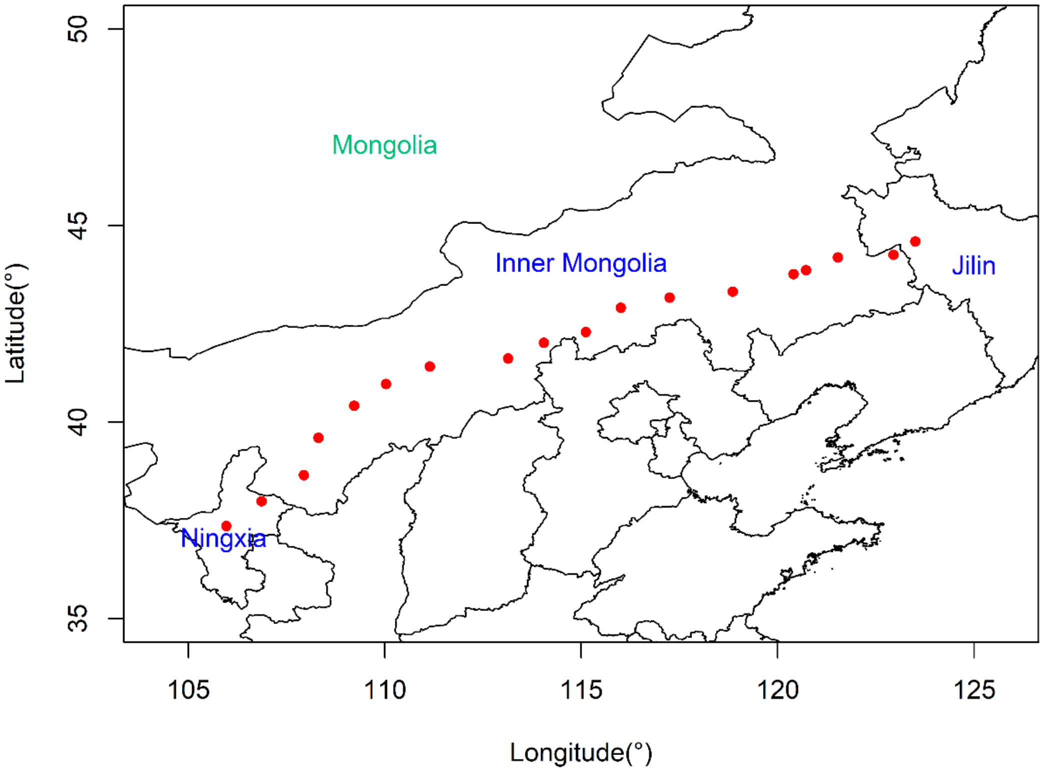

Figure S2 Map of study region and sampling locations across the semi-arid grasslands of northern China

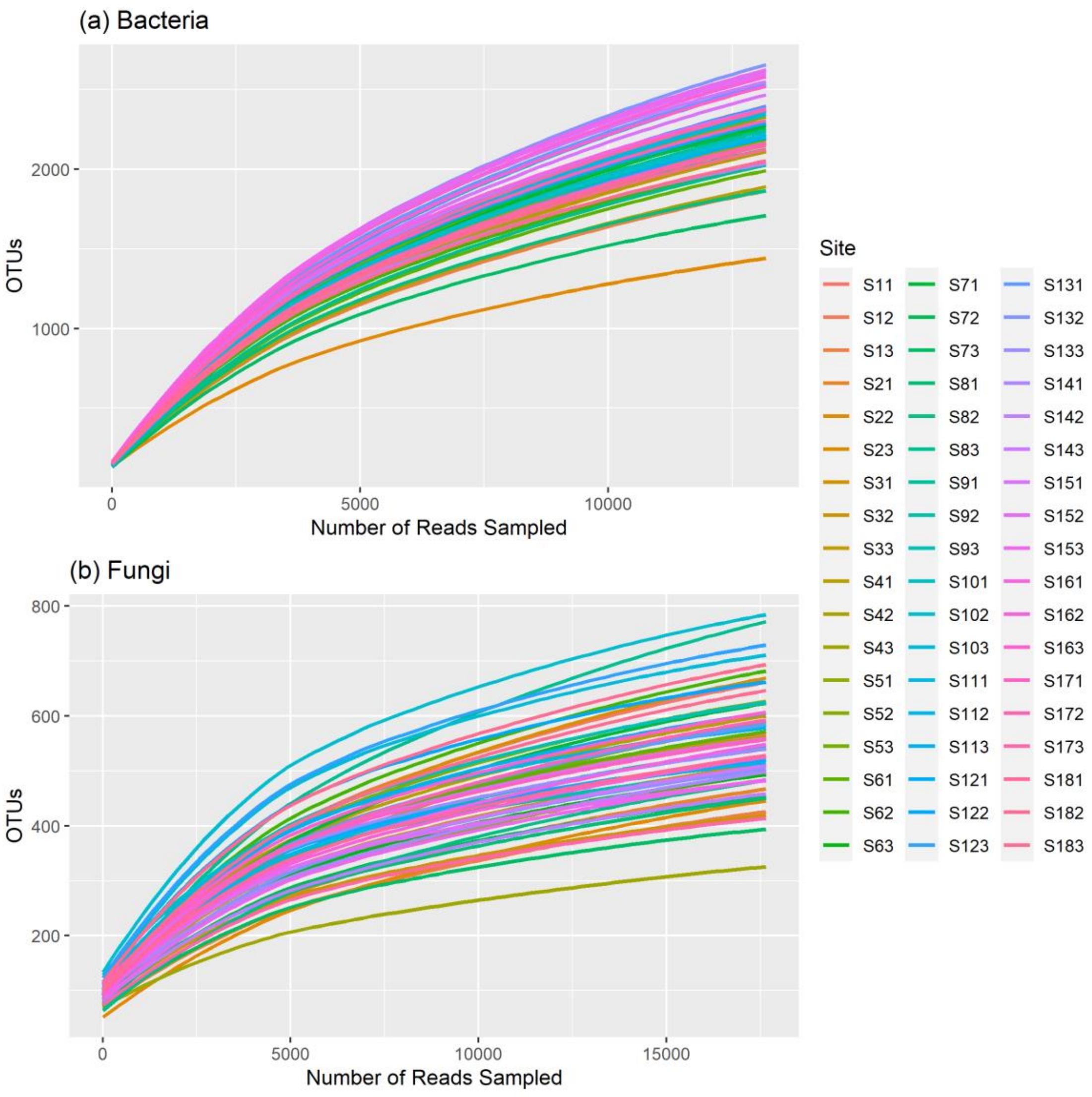

Figure S3 The rarefaction curves of soil bacterial (a) and fungal (b) samples across 54 sites in semi-arid grasslands of northern China.

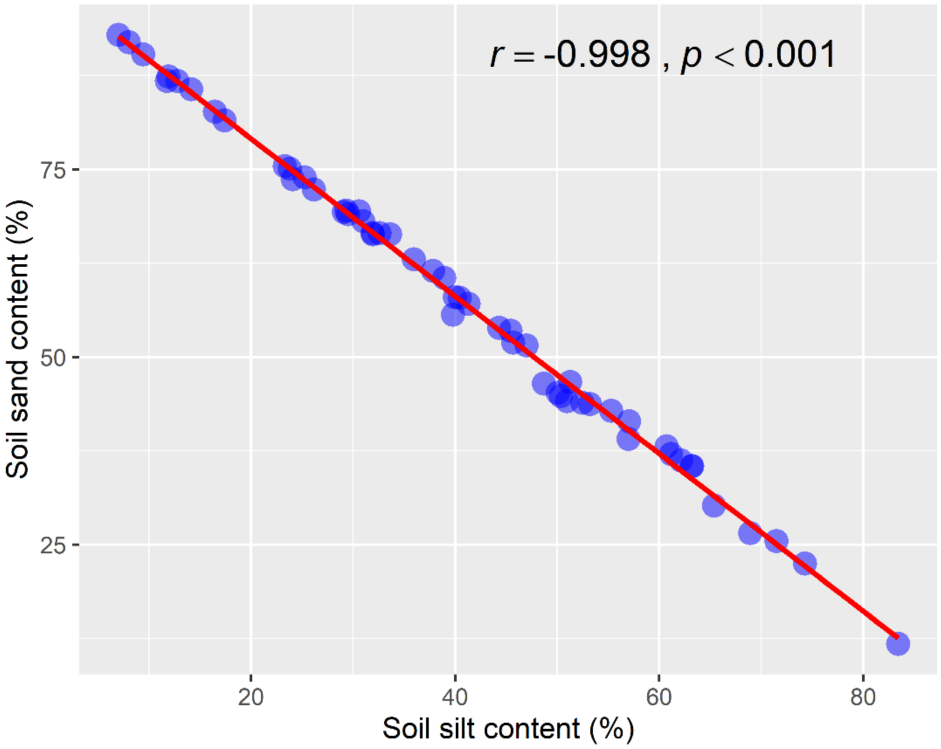

Figure S4 The relationship between soil silt content and soil sand content. The red line represents significant correlation at $p < 0.05$.
